# Supplementary material for: ACAP1 Deficiency Predicts Inferior Immunotherapy Response in Solid Tumors
Source: Cancers (Basel). 2022 Dec 1;14(23):5951. doi: 10.3390/cancers14235951 (PMC9740925; doi:10.3390/cancers14235951)
Supplement: Supplementary file 1 [file cancers-14-05951-s001.zip › Supplementary figure legends and table S1.pdf]

Supplementary Material:

Table S1. Abbreviation

**TCGA cancer types abbreviations**	
**Abbreviation**	**Full name**
ACC	Adrenocortical carcinoma
BLCA	Bladder Urothelial Carcinoma
BRCA	Breast invasive carcinoma
CESC	Cervical squamous cell carcinoma and endocervical adenocarcinoma
CHOL	Cholangiocarcinoma
COAD	Colon adenocarcinoma
DLBC	Lymphoid Neoplasm Diffuse Large B-cell Lymphoma
ESCA	Esophageal carcinoma
GBM	Glioblastoma multiforme
HNSC	Head and Neck squamous cell carcinoma
KICH	Kidney Chromophobe
KIRC	Kidney renal clear cell carcinoma
KIRP	Kidney renal papillary cell carcinoma
LAML	Acute Myeloid Leukemia
LGG	Brain Lower Grade Glioma
LIHC	Liver hepatocellular carcinoma
LUAD	Lung adenocarcinoma
LUSC	Lung squamous cell carcinoma
MESO	Mesothelioma
OV	Ovarian serous cystadenocarcinoma
PAAD	Pancreatic adenocarcinoma
PCPG	Pheochromocytoma and Paraganglioma
PRAD	Prostate adenocarcinoma
READ	Rectum adenocarcinoma
SARC	Sarcoma
SKCM	Skin Cutaneous Melanoma
STAD	Stomach adenocarcinoma
TGCT	Testicular Germ Cell Tumors
THCA	Thyroid carcinoma
THYM	Thymoma
UCEC	Uterine Corpus Endometrial Carcinoma
UCS	Uterine Carcinosarcoma
UVM	Uveal Melanoma
**Other abbreviations**	

Abbreviation	Full name
ACAP1	ArfGAP With Coiled-Coil, Ankyrin Repeat And PH Domains 1
AUC	Area under the ROC curve
ccRCC	Clear cell renal cell carcinoma
RCC	Renal cell carcinoma
CGGA	Chinese Glioma Genome Atlas
CENTB1	Centaurin beta1
CNV	Copy Number Variation
CNA	Copy Number Alteration
CPTAC	Clinical Proteomic Tumor Analysis Consortium
CYT	Cytotoxicity score
DFI	Disease-Free Interval
DSS	Disease-Specific Survival
GEO	Gene Expression Omnibus
GTEx	Genotype-Tissue Expression Project
HCC	Hepatocellular carcinoma
ICGC	International Cancer Genome Consortium
ICT	Immune checkpoint blockade therapy
MDSC	Myeloid derived suppressor cell
MSI	Microsatellite Instability
mUC	metastatic urothelial cancer
NSCLC	Non-Small Cell Lung Cancer
OS	Overall Survival
PFI	Progression-Free Interval
PFS	Progression-Free Survival
ROC	Receiver Operating Characteristic
RPKM	Reads per kilo base per million mapped reads
TCGA	The Cancer Genome Atlas
TIDE	Tumor Immune Dysfunction and Exclusion
TILs	Tumor-infiltrating lymphocytes
TMB	Tumor Mutational Burden
TPM	Transcripts Per Million
tSNE	t-distributed stochastic neighbor embedding

Supplementary figure legends:

Figure S1. ACAP1 expression levels in mouse tissues and cell lines were visualized by BioGPS.

Figure S2. Stacked bar graphs illustrate the proportion of cells with different ACAP1 levels in various cell types in the GSE72096 and GSE115978 datasets, respectively.

Figure S3. The ACAP1 expression across different cell types in single-cell sequencing datasets was represented as violin plots, respectively.

Figure S4. Differential expression of ACAP1 in different T stages across TCGA tumor types.

Figure S5. Differential expression of ACAP1 in different N stages across TCGA tumor types.

Figure S6. Differential expression of ACAP1 in different M stages across TCGA tumor types.

Figure S7. Differential expression of ACAP1 in different pathologic or clinical stages across TCGA tumor types.

Figure S8. Disease-specific survival analyses of cancer patients stratified by ACAP1 mRNA level with Kaplan-Meier method in TCGA datasets.

Figure S9. Progression-free interval analyses of cancer patients stratified by ACAP1 mRNA level with Kaplan-Meier method in TCGA datasets.

Figure S10. Genomic alterations of ACAP1 across TCGA cancer types. The prevalence of ACAP1 genomic aberrations across cancer types in TCGA. CNA , copy number alteration.

Figure S11. GSEA analyses of the co-expressed genes of ACAP1 across 33 cancer types in TCGA. The top five significant “biological processes” correlated with ACAP1 expression were plotted for each cancer type.

Figure S12. The spearman correlation of ACAP1 expression with infiltration level of dendritic cell, monocyte, macrophage, mast cell, neutrophil, eosinophil, Granulocyte-monocyte progenitor, Common myeloid progenitor, and Common lymphoid progenitor using the TIMER2 web tool.

Figure S13. The Spearman correlations of ACAP1 expression and immune-related gene expression across 33 cancer types in TCGA. (A) Correlation of ACAP1 expression with cytotoxic genes (GZMA and PRF1) expression and cytotoxicity score (CYT). (B) The correlation of ACAP1 expression with MHC gene expression.

Figure S14. Pan-cancer analysis of the correlation between ACAP1 expression and TMB (A) or MSI (B) across 33 cancer types in TCGA.
