# Supplementary figures and images for: ACAP1 Deficiency Predicts Inferior Immunotherapy Response in Solid Tumors
Source: Cancers (Basel). 2022 Dec 1;14(23):5951. doi: 10.3390/cancers14235951 (PMC9740925; doi:10.3390/cancers14235951)

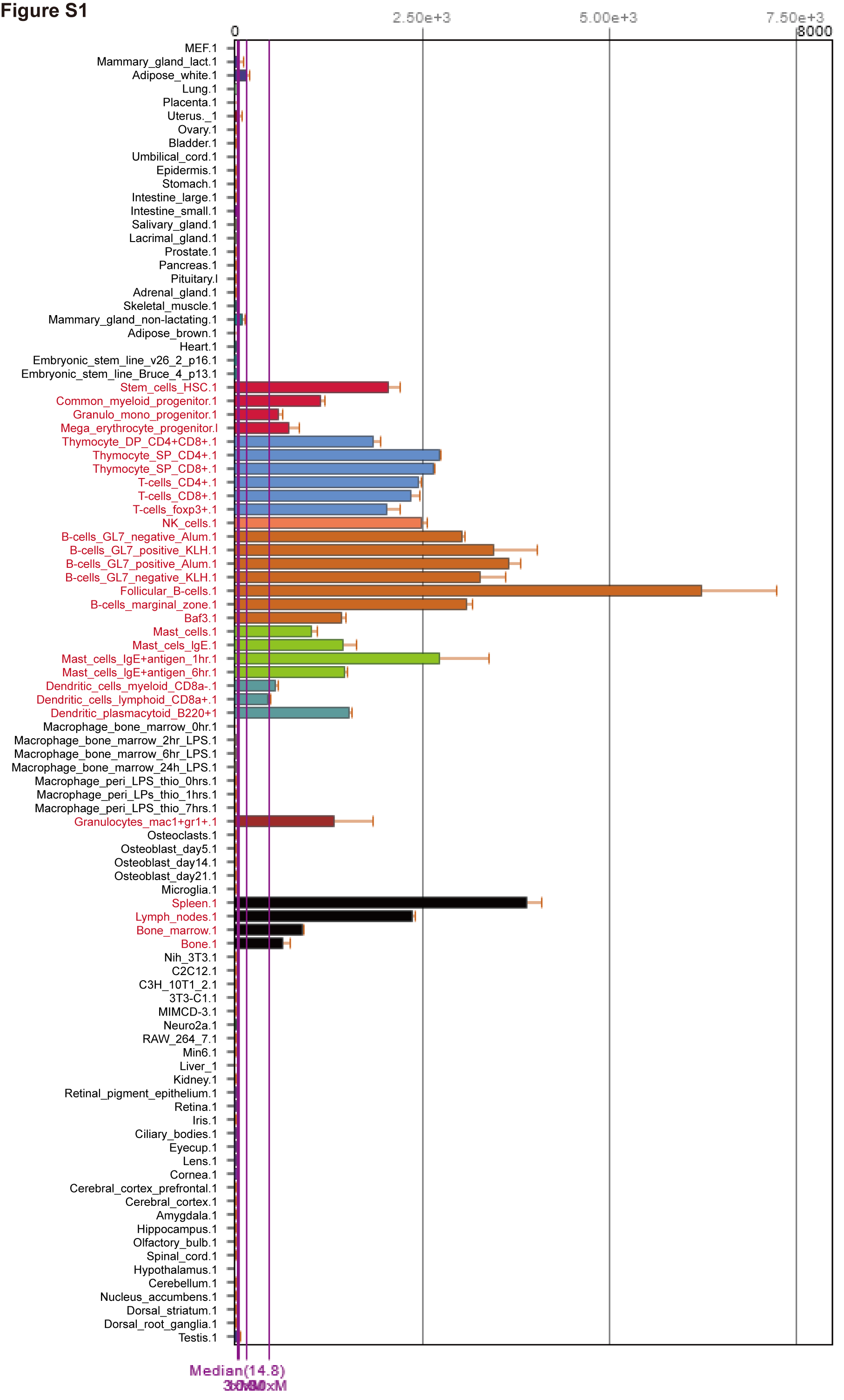

Supplement: Supplementary file 1 [file cancers-14-05951-s001.zip › Figure S1.tif]

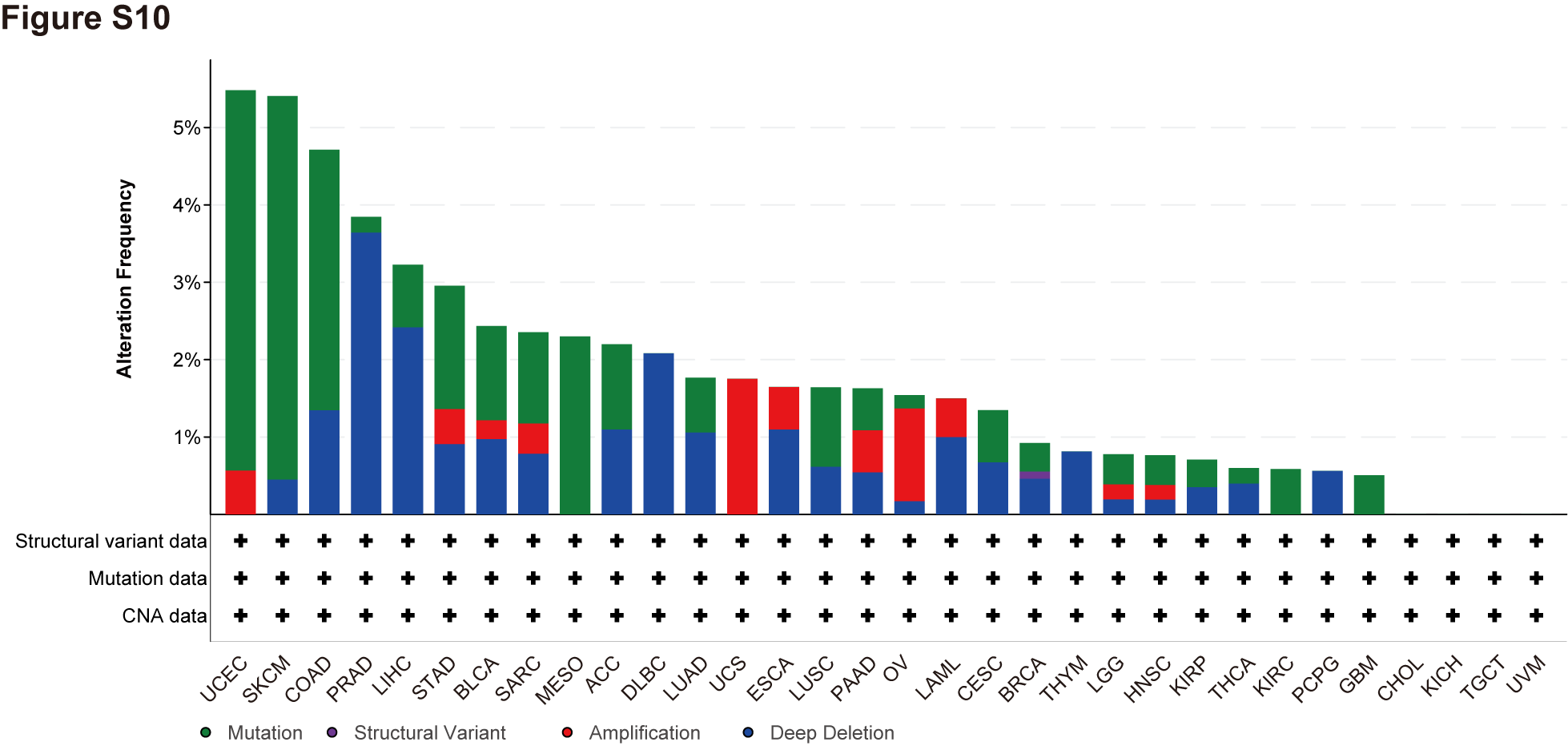

Supplement: Supplementary file 1 [file cancers-14-05951-s001.zip › Figure S10.tif]

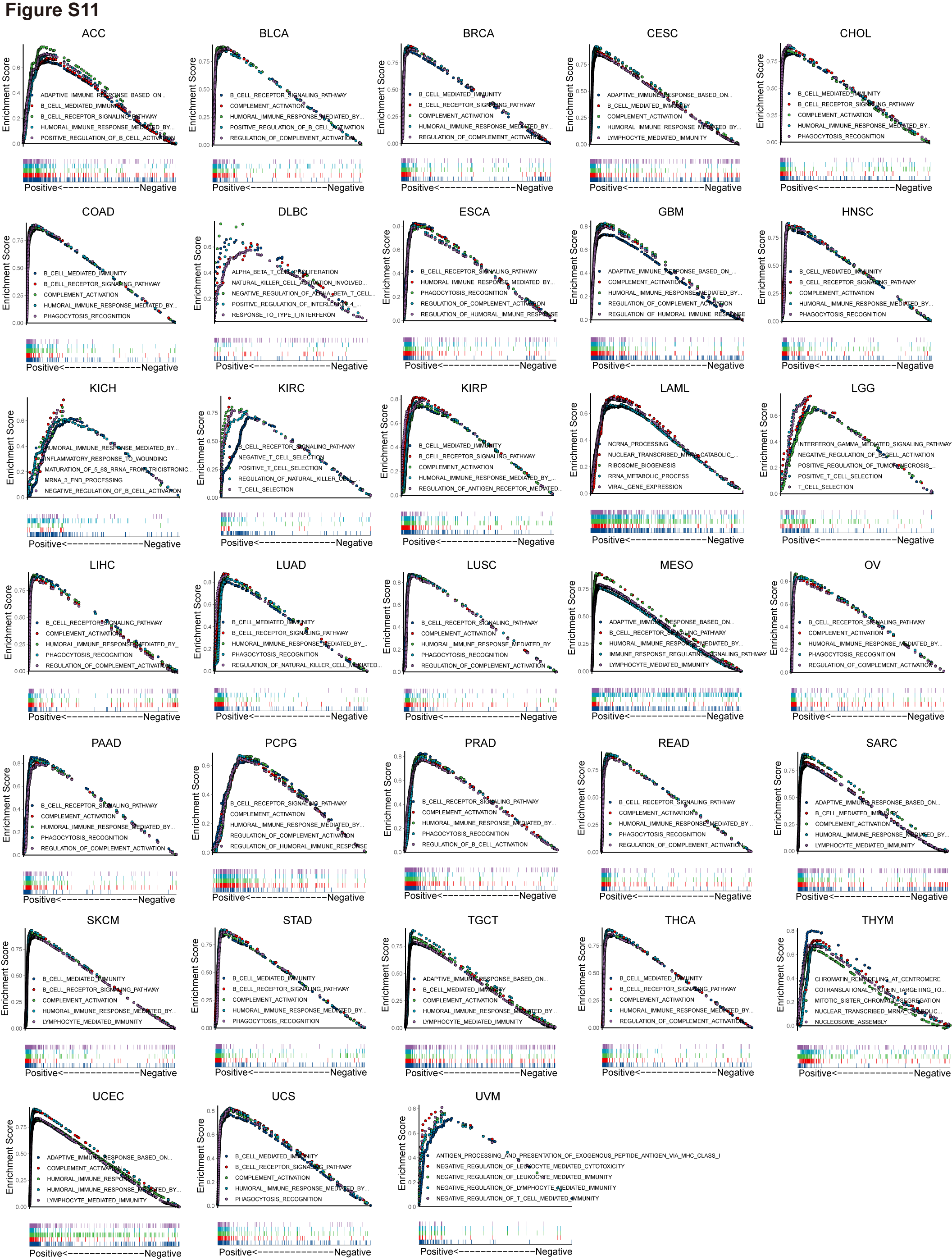

Supplement: Supplementary file 1 [file cancers-14-05951-s001.zip › Figure S11.tif]

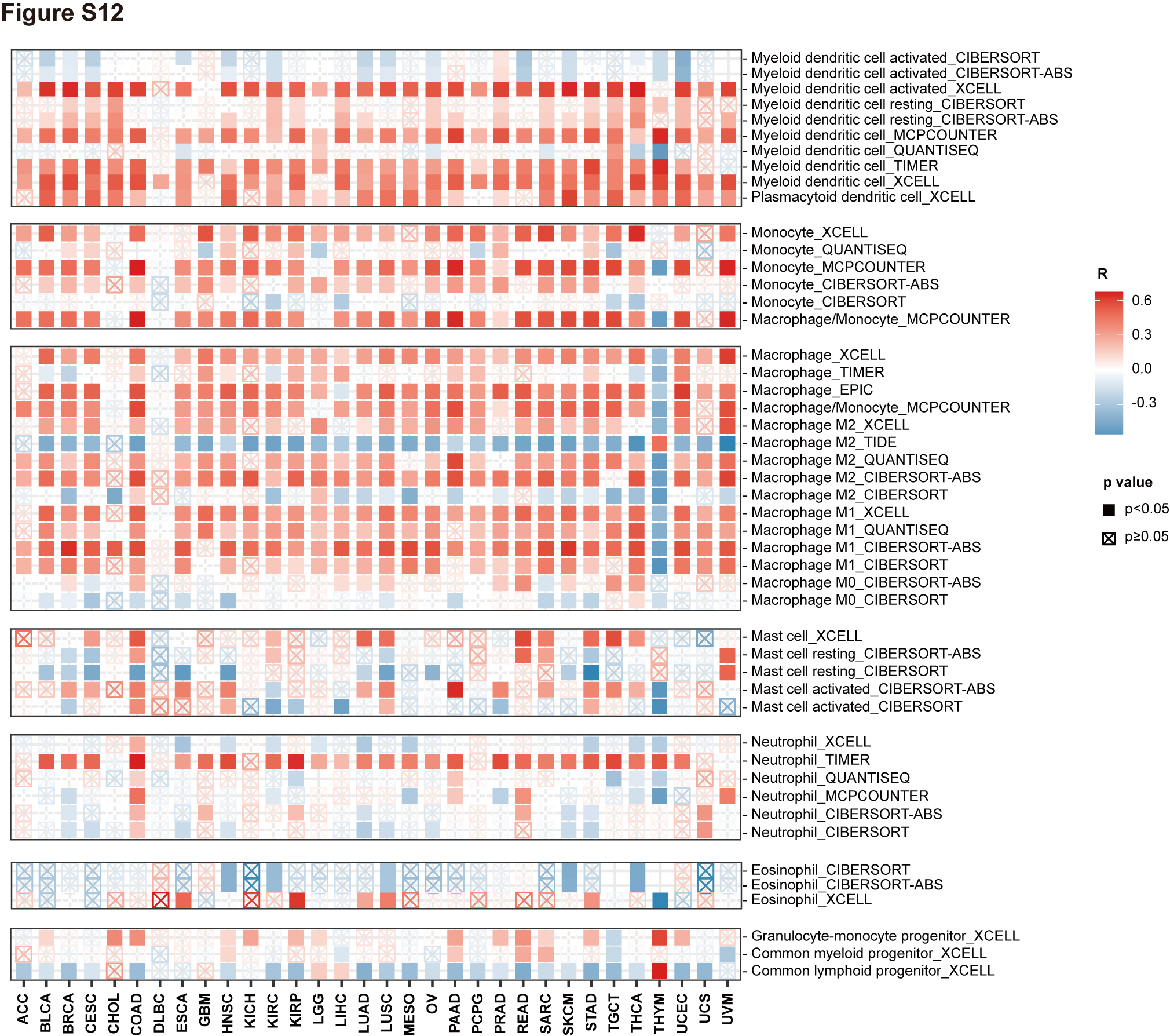

Supplement: Supplementary file 1 [file cancers-14-05951-s001.zip › Figure S12.tif]

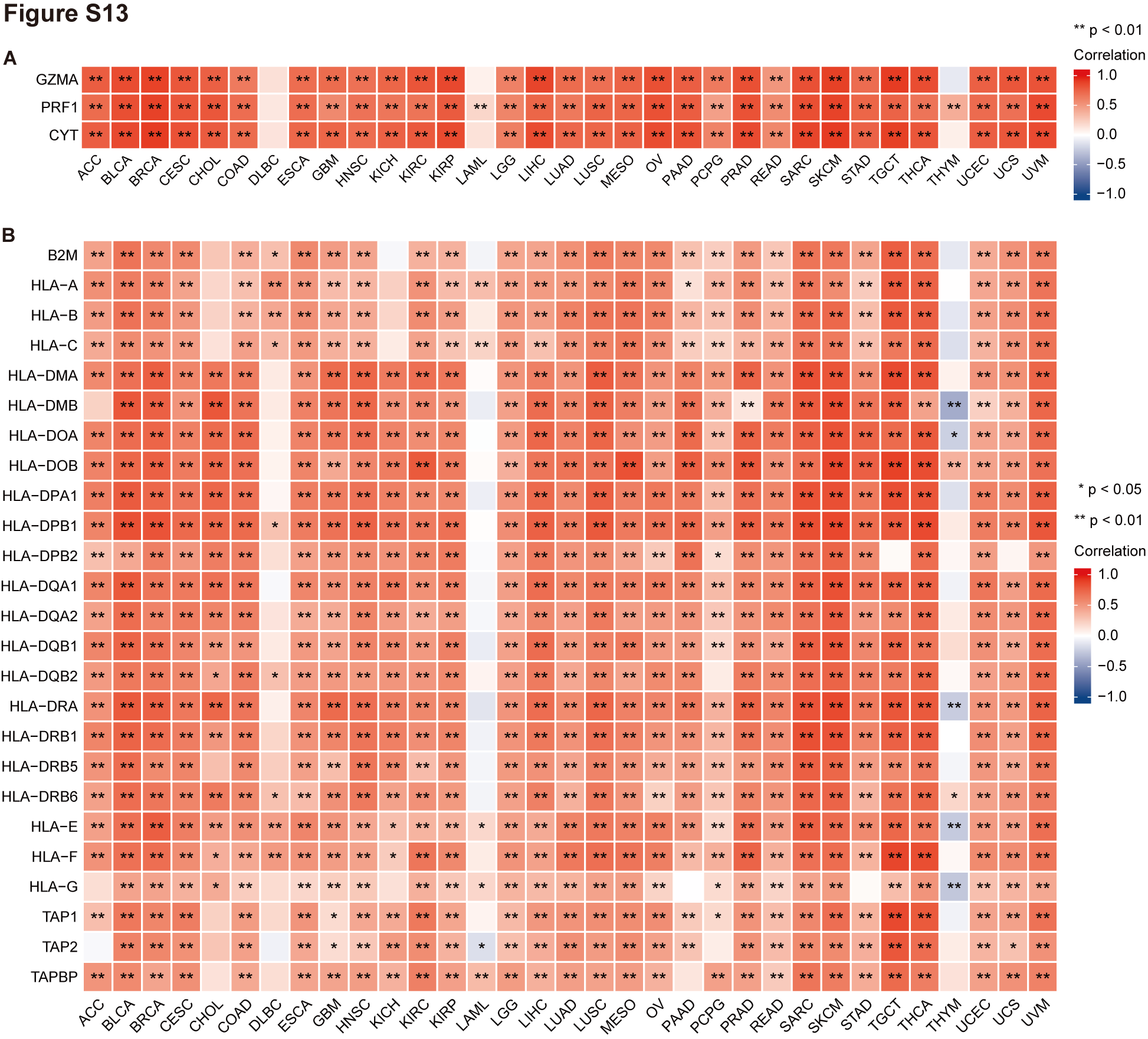

Supplement: Supplementary file 1 [file cancers-14-05951-s001.zip › Figure S13.tif]

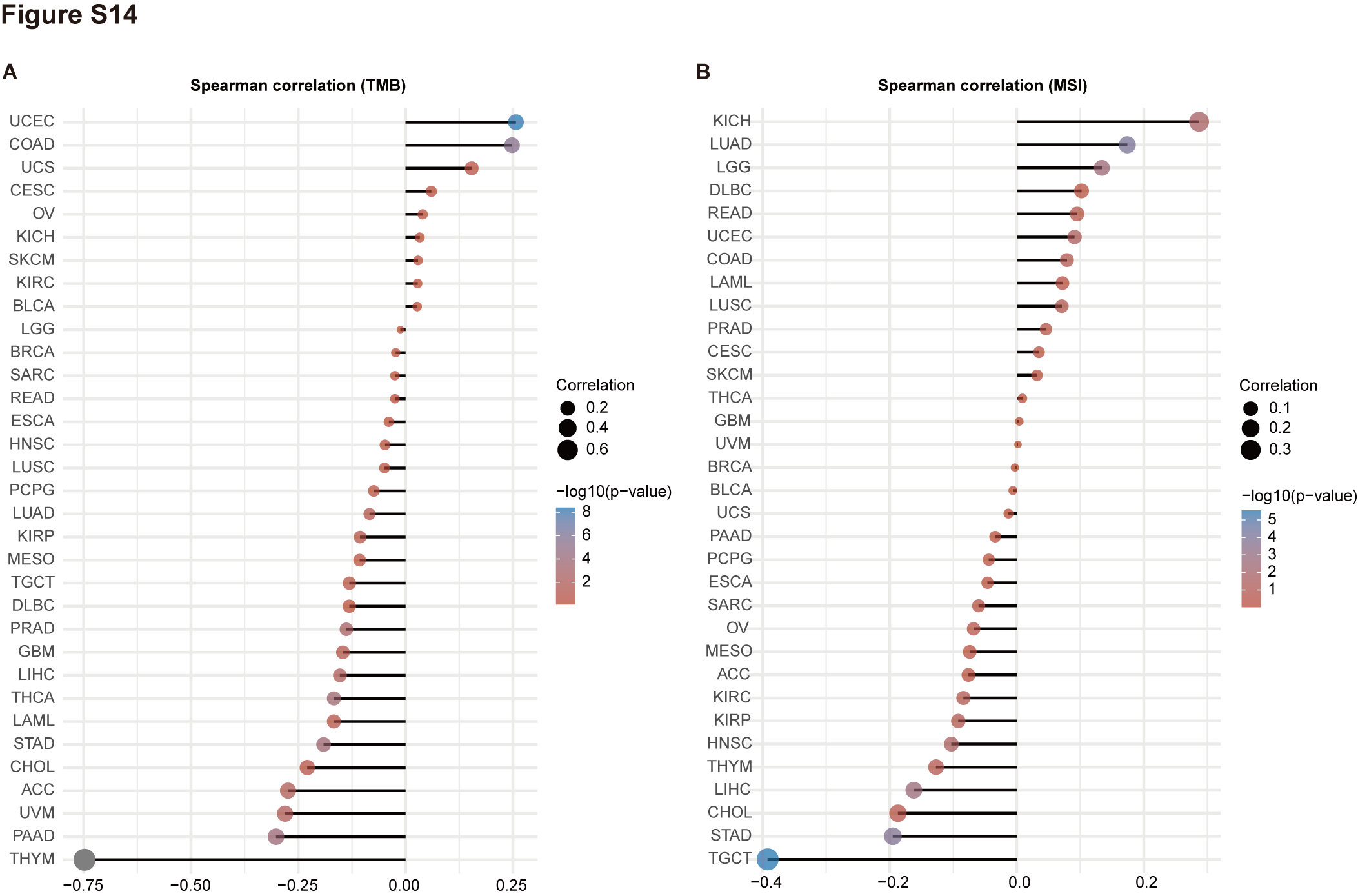

Supplement: Supplementary file 1 [file cancers-14-05951-s001.zip › Figure S14.tif]

Figure 1D

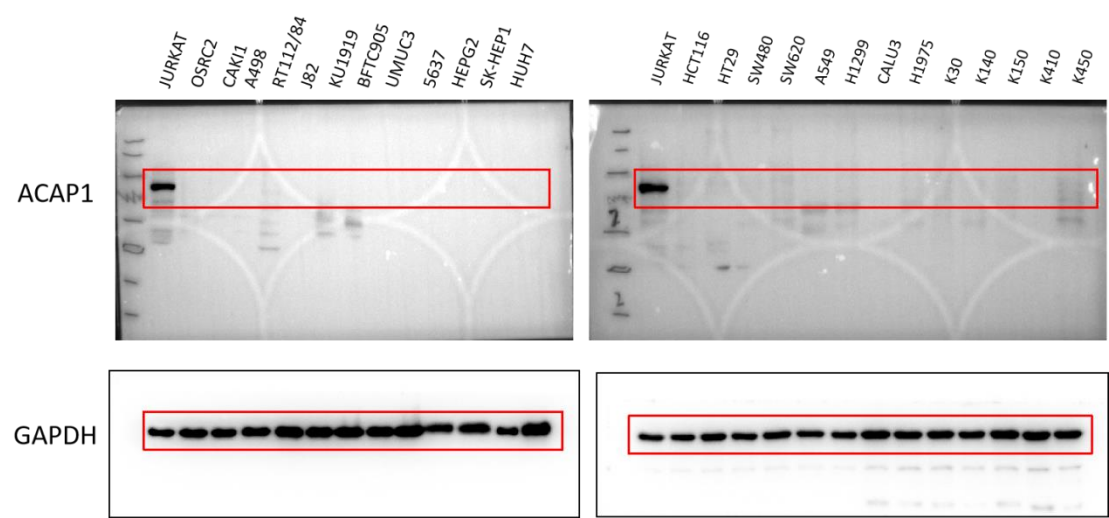

Figure 6G

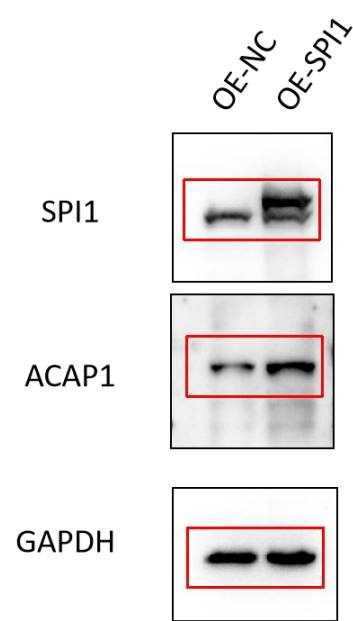

**Figure 6H**

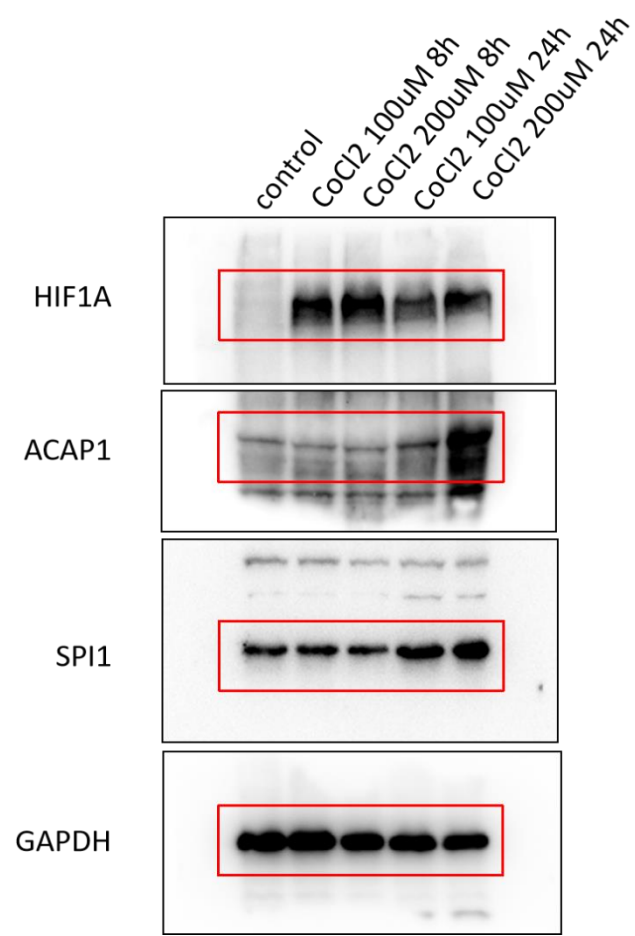

**Figure 8A**

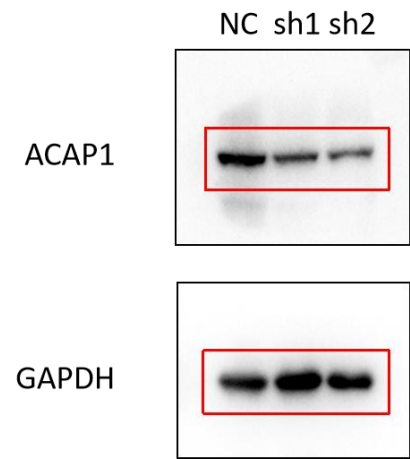

Supplement: Supplementary file 1 [file cancers-14-05951-s001.zip › Figure S15. original WB images.pdf]

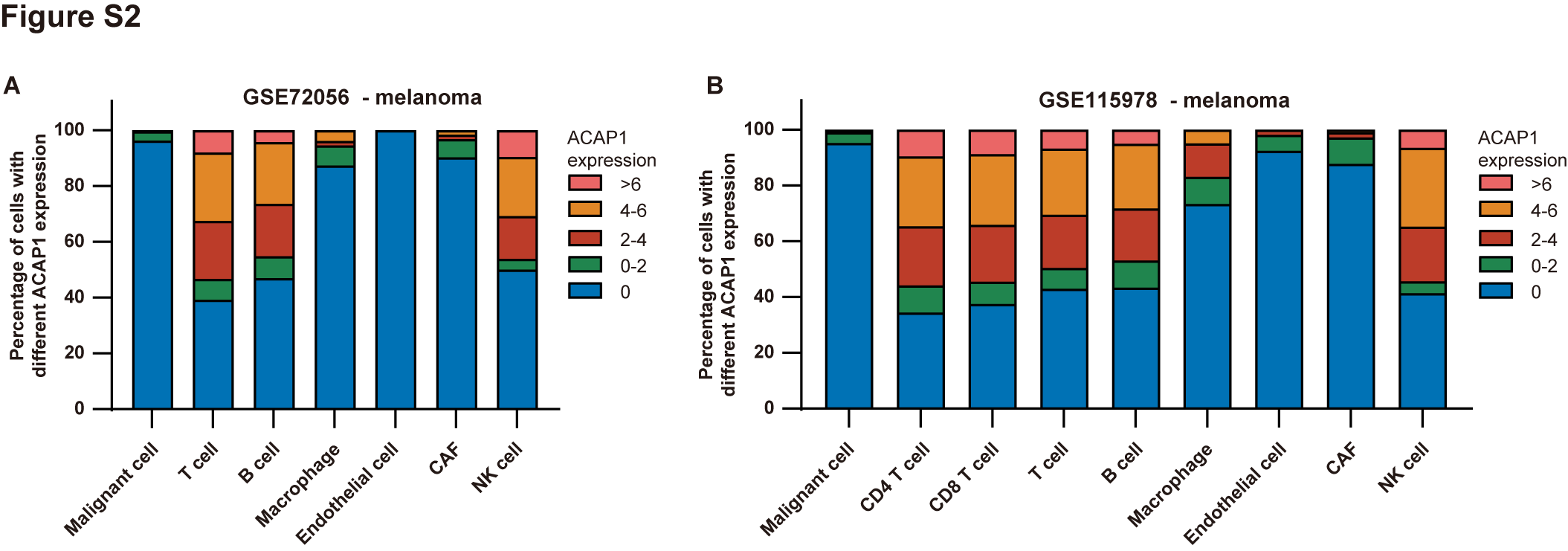

Supplement: Supplementary file 1 [file cancers-14-05951-s001.zip › Figure S2.tif]

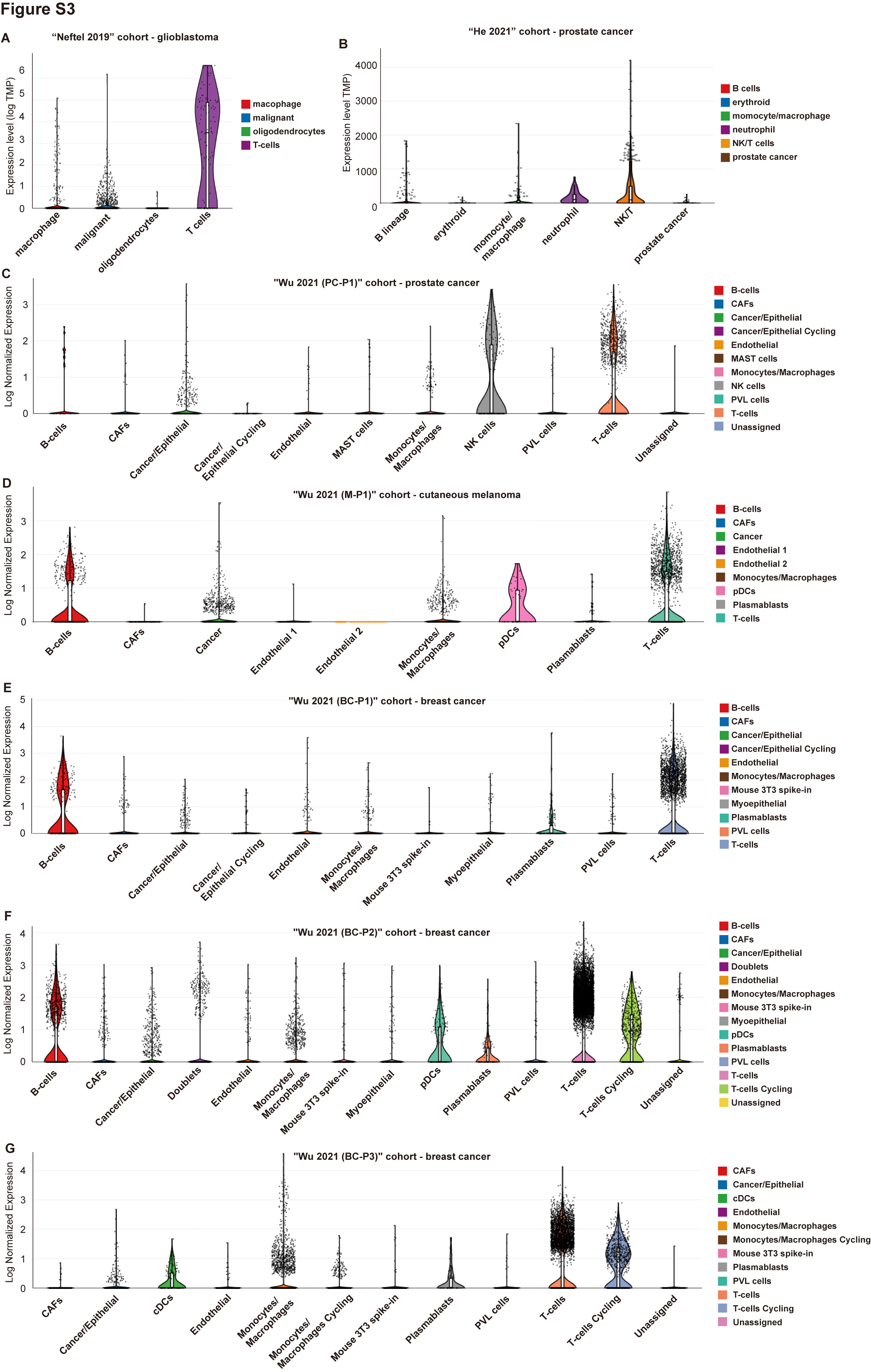

Supplement: Supplementary file 1 [file cancers-14-05951-s001.zip › Figure S3.tif]

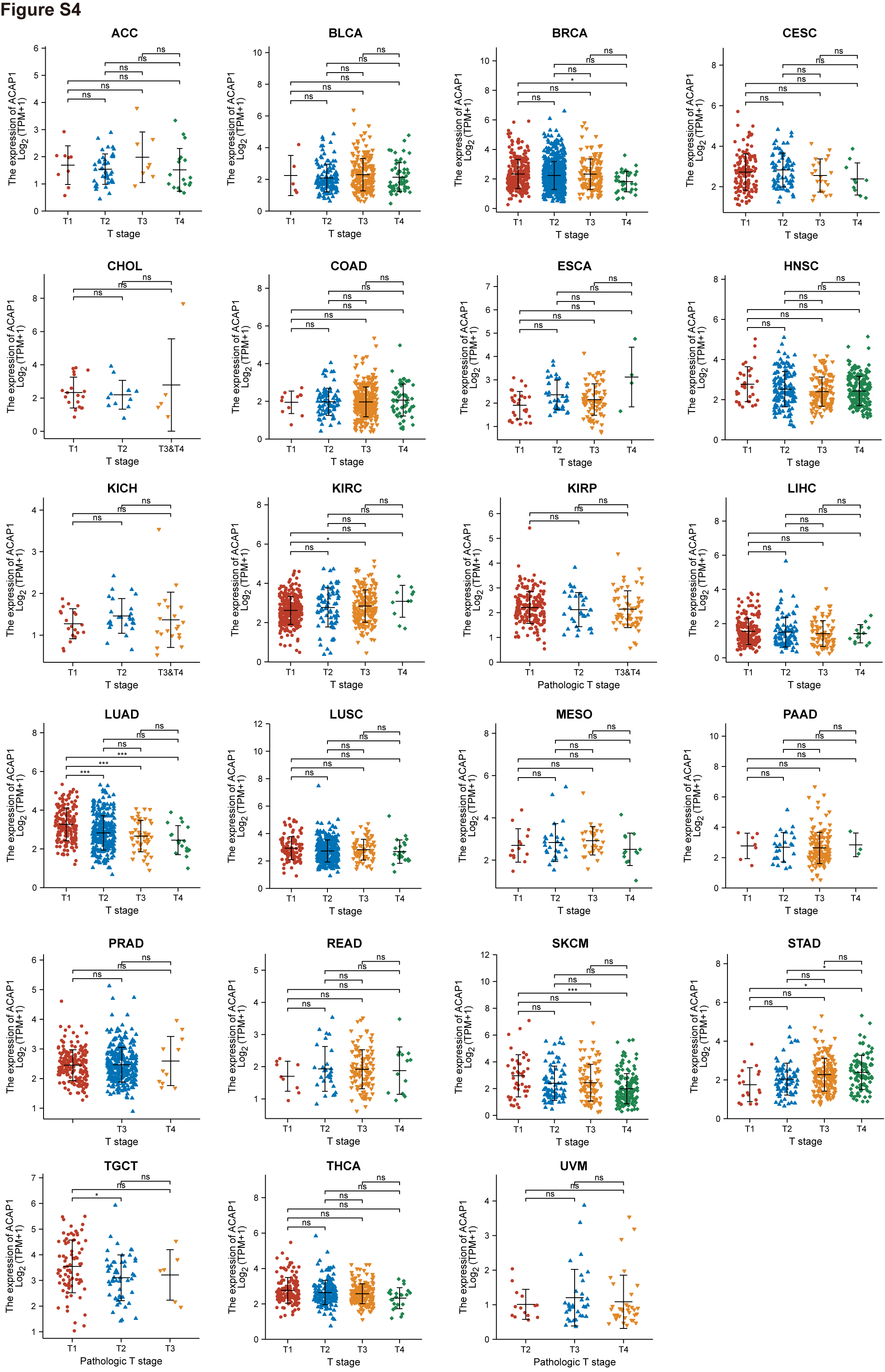

Supplement: Supplementary file 1 [file cancers-14-05951-s001.zip › Figure S4.tif]

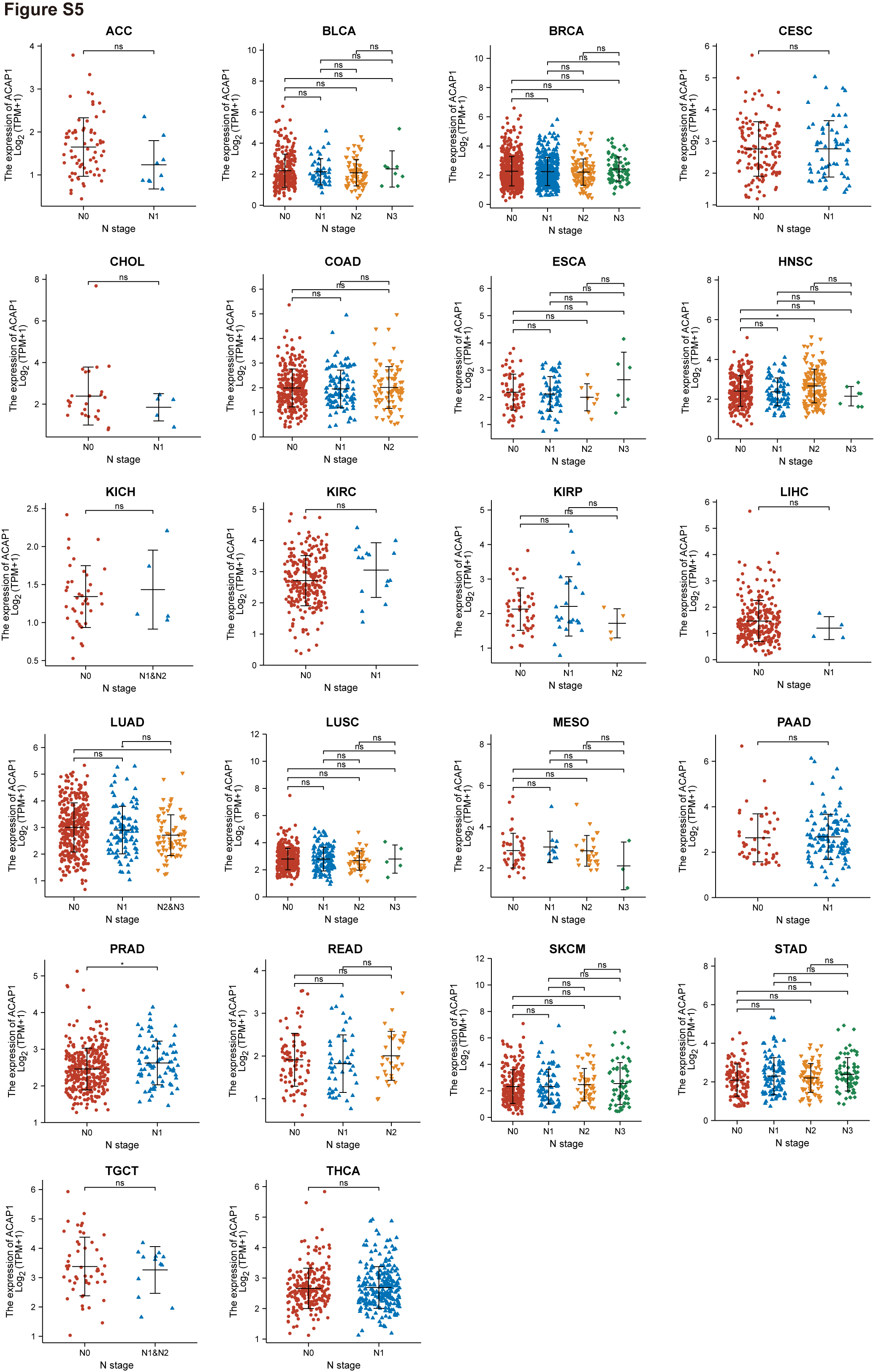

Supplement: Supplementary file 1 [file cancers-14-05951-s001.zip › Figure S5.tif]

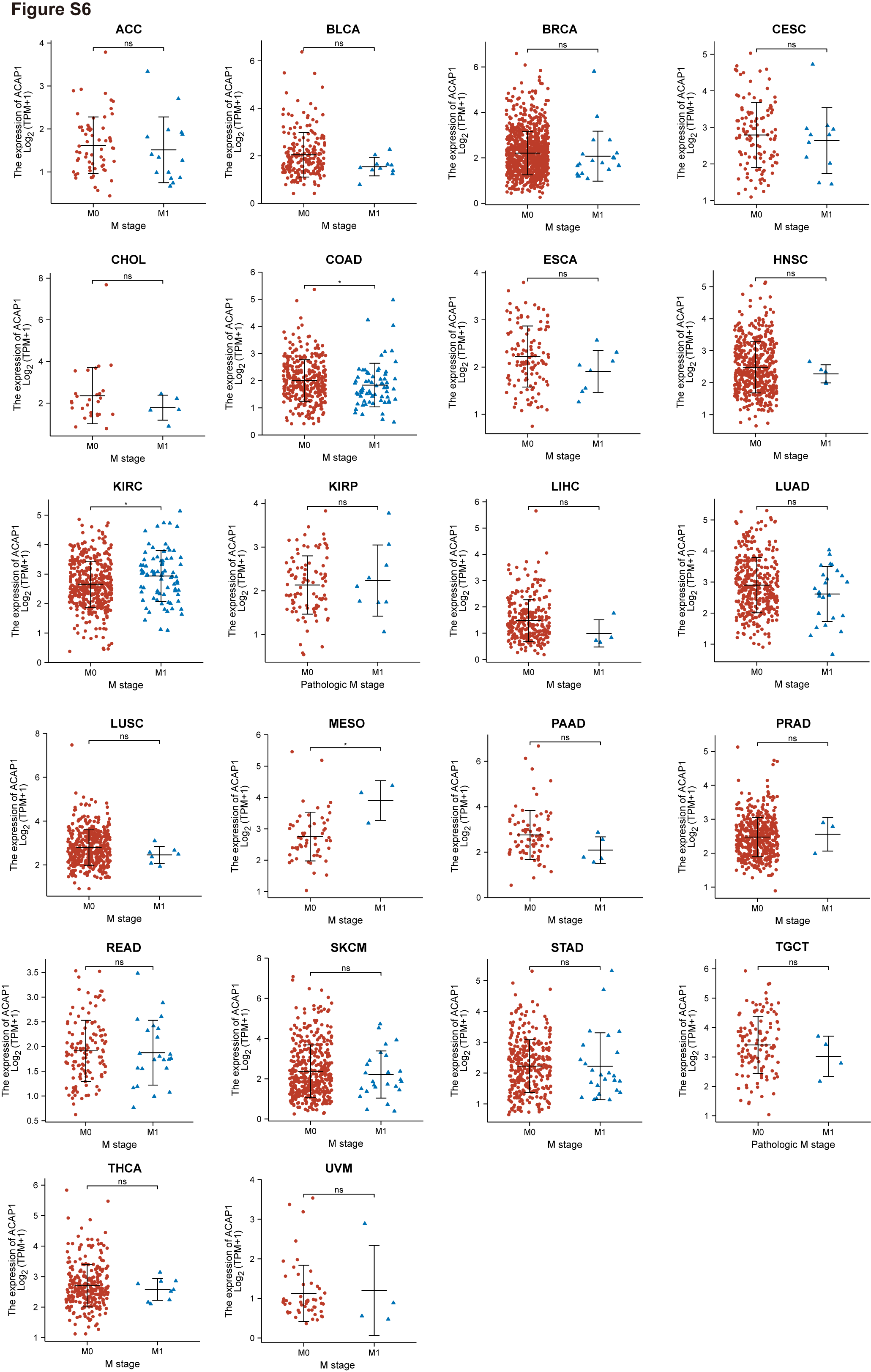

Supplement: Supplementary file 1 [file cancers-14-05951-s001.zip › Figure S6.tif]

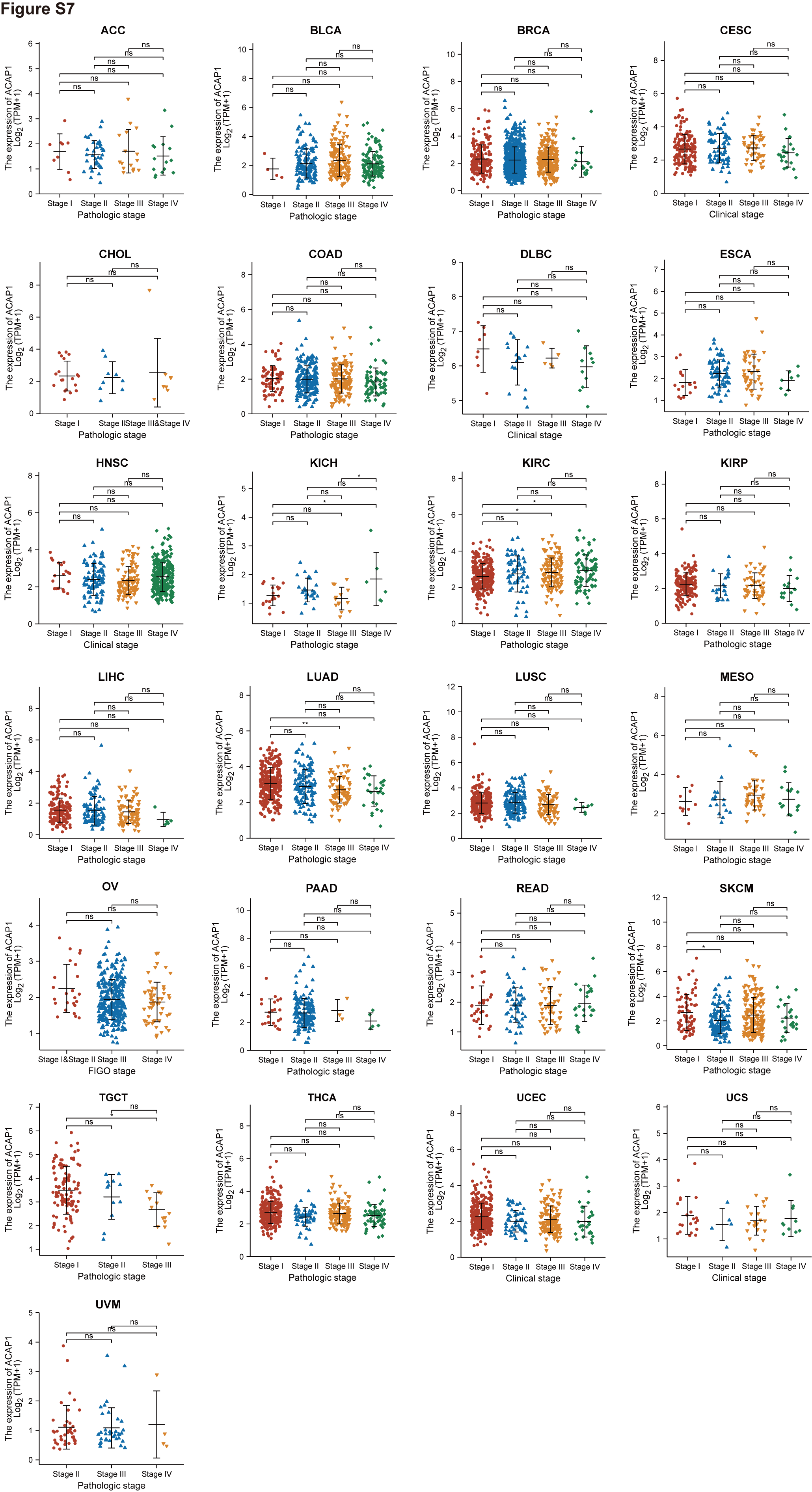

Supplement: Supplementary file 1 [file cancers-14-05951-s001.zip › Figure S7.tif]

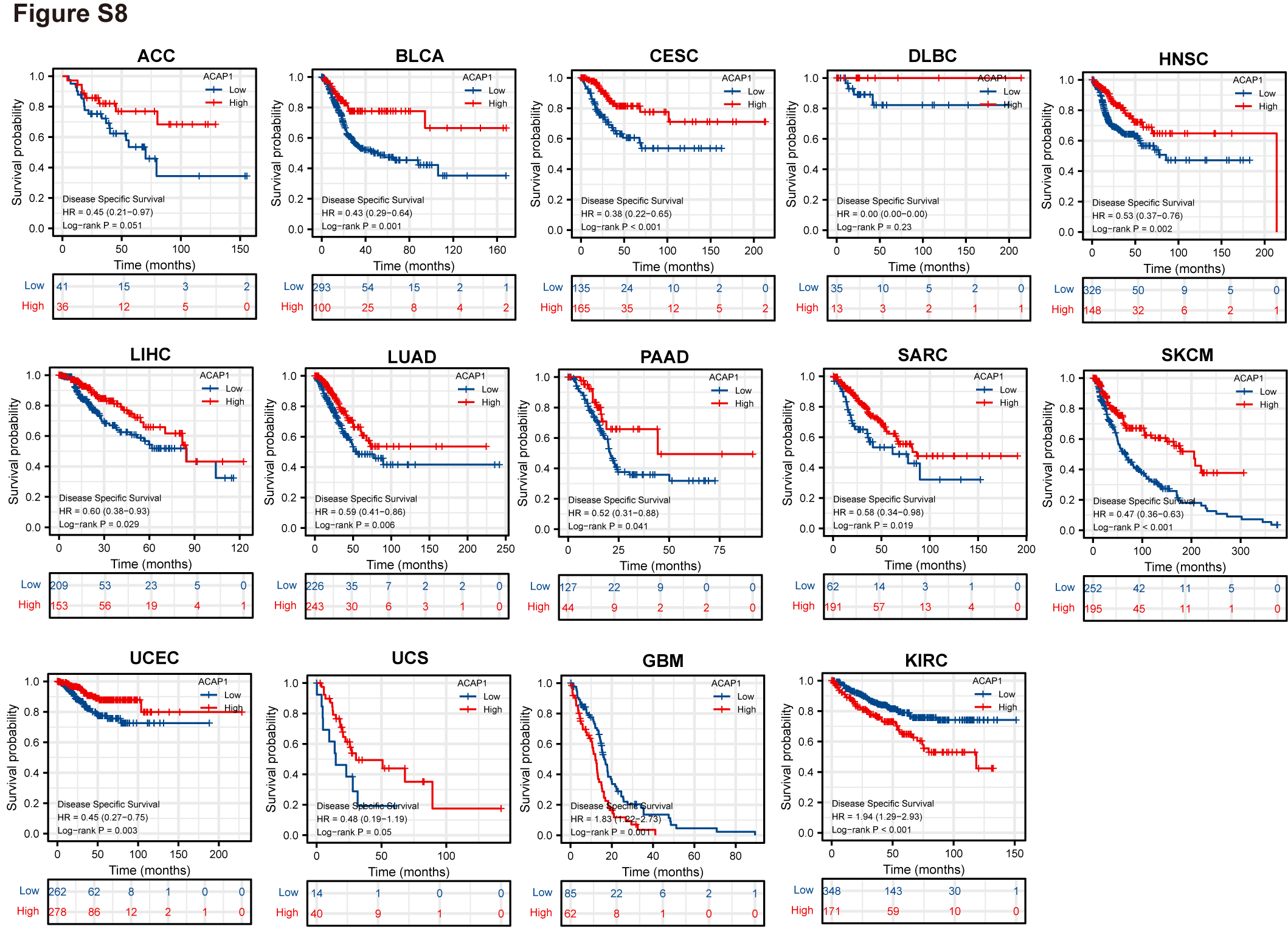

Supplement: Supplementary file 1 [file cancers-14-05951-s001.zip › Figure S8.tif]

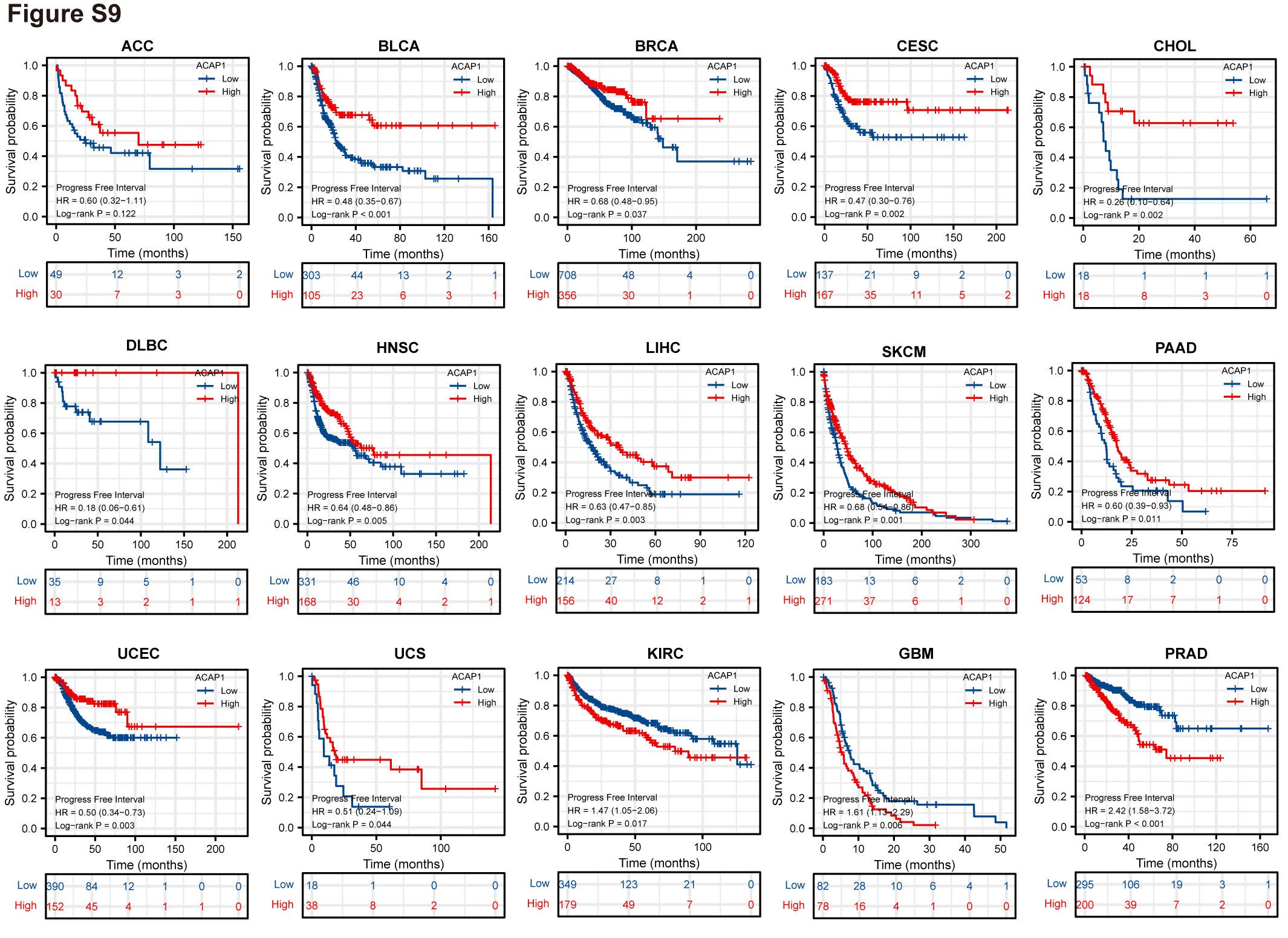

Supplement: Supplementary file 1 [file cancers-14-05951-s001.zip › Figure S9.tif]
